# *anti*-1′,6′,7′,8′,9′,14′,15′,16′-Octa­chloro­dispiro­[1,3-dioxolane-2,17′-penta­cyclo­[12.2.1.1^6,9^.0^2,13^.0^5,10^]octa­decane-18′,2′′-1,3-dioxolane]-7′,15′-diene

**DOI:** 10.1107/S1600536810024669

**Published:** 2010-07-03

**Authors:** Megan E. Tenbusch, Matthew D. Brooker, Jacob C. Timmerman, Daniel S. Jones, Markus Etzkorn

**Affiliations:** a Department of Chemistry, The University of North Carolina at Charlotte, 9201 University City Blvd, Charlotte, NC 28223, USA

## Abstract

The title compound, C_22_H_20_Cl_8_O_4_, was prepared as part of the synthesis of precursors for the preparation of fluorinated mol­ecular tweezers. The mol­ecule sits on an inversion center, thus requiring that the cyclo­octane ring adopt a chair conformation.

## Related literature

For related structures, see: Garcia *et al.* (1991*b* ▶,*c* ▶). For related chemistry on analogous polycyclic scaffolds, see: Garcia *et al.* (1991*a* ▶); Chou *et al.* (2005 ▶)
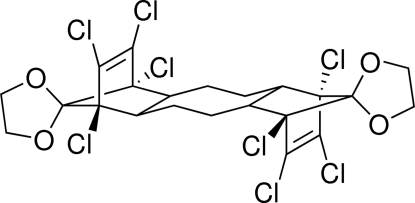


## Experimental

### 

#### Crystal data

C_22_H_20_Cl_8_O_4_
*M* *_r_* = 631.98
Monoclinic, 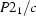

*a* = 9.5332 (7) Å
*b* = 7.9121 (6) Å
*c* = 17.014 (2) Å
β = 101.099 (8)°
*V* = 1259.3 (2) Å^3^
*Z* = 2
Cu *K*α radiation
μ = 8.44 mm^−1^
*T* = 295 K
0.25 × 0.20 × 0.08 mm

#### Data collection

Enraf–Nonius CAD-4 diffractometer
Absorption correction: multi-scan (Blessing, 1995 ▶) *T*_min_ = 0.190, *T*_max_ = 0.561
4703 measured reflections
2275 independent reflections
1702 reflections with *I* > 2σ(*I*)
*R*_int_ = 0.047
3 standard reflections every 62 reflections  intensity decay: 13%

#### Refinement

*R*[*F* ^2^ > 2σ(*F* ^2^)] = 0.041
*wR*(*F* ^2^) = 0.118
*S* = 1.05
2275 reflections
155 parameters
H-atom parameters constrained
Δρ_max_ = 0.36 e Å^−3^
Δρ_min_ = −0.47 e Å^−3^

### 

Data collection: *CAD-4 EXPRESS* (Enraf–Nonius, 1994 ▶); cell refinement: *CAD-4 EXPRESS*; data reduction: *XCAD4* (Harms & Wocadlo, 1995 ▶); program(s) used to solve structure: *DIRDIF08* (Beurskens *et al.*, 2008 ▶); program(s) used to refine structure: *SHELXL97* (Sheldrick, 2008 ▶); molecular graphics: *ORTEP-3 for Windows* (Farrugia, 1997 ▶); software used to prepare material for publication: *WinGX* (Farrugia, 1999 ▶).

## Supplementary Material

Crystal structure: contains datablocks global, I. DOI: 10.1107/S1600536810024669/jh2171sup1.cif

Structure factors: contains datablocks I. DOI: 10.1107/S1600536810024669/jh2171Isup2.hkl

Additional supplementary materials:  crystallographic information; 3D view; checkCIF report

## Figures and Tables

**Fig. 1.**
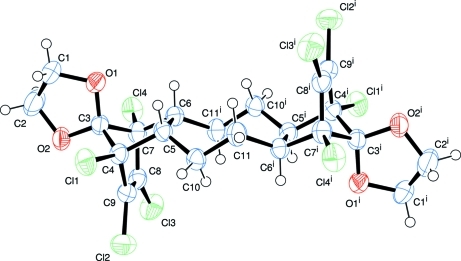
A view of the title compound with 50% probability displacement ellipsoids. [Symmetry code: (i) -x + 2, -y + 2, -z + 2]

**Fig. 2.**
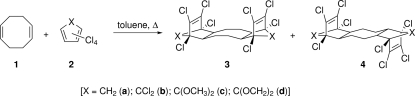
Synthesis scheme.

## supplementary crystallographic information

### Comment

The twofold Diels-Alder reaction of cyclooctadiene 1 with two equivalents of cyclopentadiene or cyclopentadienone derivatives (2a-c) furnishes the corresponding polycyclic bisadducts *endo,endo,syn*-3 and *endo,endo,anti*- 4 in a 1:4 ratio (Garcia *et *al*.*, 1991*a,b,c*)*. *For the synthesis of compounds with new luminescent properties (Chou *et al.*, 2005) or the construction of molecular tweezers *syn* derivative 3 is an ideal starting material with the required orientation of both double bonds on one side of the molecule. Nevertheless, the separation of *syn* isomer 3c from *anti* ketal 4c prior to subsequent functionalization was often unsatisfactory in our hands. Thus, we converted cyclooctadiene 1 with the spiroketal 2 d to the spiropolycyclic bisadducts 3 d and 4 d in 85–90% yield, typically with an isomer distribution that did not differ significantly from the non-spirocyclic ketal case (1+2c). Furthermore, compound 3 d was easily separated from *anti*-isomer 4 d by repeated recrystallization from hot diethyl ether, *i.e.*, the ether solution becomes more enriched in *syn*-isomer 3 d, and initially the clean *anti*-isomer 4 d precipitates upon cooling. We were able to grow single crystals of 4 d from chloroform and determined the crystal structure of compound 4 d, thus confirming the correct spectroscopic assignment of both isomers.

Two closely related structures have been found. The first (Garcia *et al., 1*991*b*) has an open ketal structure on each of the bridgehead carbon atoms, while the second (Garcia *et al., 1*991*c*) has no substituents on the bridgehead carbon atoms. Each of these two structures sits on an inversion center and thus assumes a conformation nearly identical to that of the title compound.

### Experimental

A mixture of cyclooctadiene 1 (3 g, 29 mmol) and spiroketal 2 d (15 g, 57 mmol) was refluxed in toluene (5 ml) for three hours. The beige paste was filtered, washed with methylene chloride (70 ml), dried and washed again with methanol (*ca* 15 ml) to remove small amounts of the mono-Diels-Alder adduct. The remaining colorless solid (14.5 g, 83%) contained a 1:4 mixture of 3 d and 4 d, respectively. After one recrystallization from hot diethyl ether the pure *anti*-isomer 4 d was obtained as a colorless precipitate.

*Mp.*> 295 °C (decomposition); *IR (KBr)*: ν~ = 2952, 2905 (CH_2_),1596 (C═C), 1467 (CH_2 _deformation), 1355, 1284, 1267, 1245, 1222, 1181, 1132, 1105,1091, 1037 (C—Cl), 1009, 946, 891, 851, 809, 770, 730 cm^-1^; *^1^H NMR (CDCl_3 _; 500 MHz): *δ = 4.20–4.10 (m, 8H; H-4, -5, -4", -5"), 2.78–2.62 (m, 4H; H-2', -5', 10', -13'), 2.20–2.00 (m, 4H; H-3', -4', -11', -12'), 0.95–0.75 (m, 4H; H-3', -4', -11', -12'); *^13^C NMR (CDCL_3_,75.6 MHz): *δ=128.5 (C-7', -8', -15', -16'), 120.5 (C-17', -18'), 77.6 (C-1', -6', 9', -14'), 67.7* (C-4, -4"), 66.5* (C-5, -5"), 51.8(C-2', -5', -10', 13'), 21.9 (C-3', -4', -11', -12'); *EA:* calc. C (41.81) H (3.19); found C: 41.83, H: 3.16 (calc.).

### Refinement

H atoms were constrained using a riding model. The methylene C—H bond lengths were fixed at 0.97 Å and the methine C—H bond lengths at 0.98 Å, with *U*_iso_(H) = 1.2 U_eq._ (C).

### Crystal data

**Table:**

C_22_H_20_Cl_8_O_4_	*F*(000) = 640
*M**_r_* = 631.98	*D*_x_ = 1.677 Mg m^−^^3^
Monoclinic, *P*2_1_/*c*	Cu *K*α radiation, λ = 1.54184 Å
Hall symbol: -P 2ybc	Cell parameters from 25 reflections
*a* = 9.5332 (7) Å	θ = 5.3–18.2°
*b* = 7.9121 (6) Å	µ = 8.44 mm^−^^1^
*c* = 17.014 (2) Å	*T* = 295 K
β = 101.099 (8)°	Prism, colorless
*V* = 1259.3 (2) Å^3^	0.25 × 0.20 × 0.08 mm
*Z* = 2	

### Data collection

**Table:**

Enraf–Nonius CAD-4 diffractometer	*R*_int_ = 0.047
graphite	θ_max_ = 67.4°, θ_min_ = 4.7°
non–profiled ω/2θ scans	*h* = −11→11
Absorption correction: multi-scan (Blessing, 1995)	*k* = −9→9
*T*_min_ = 0.190, *T*_max_ = 0.561	*l* = −20→0
4703 measured reflections	3 standard reflections every 62 reflections
2275 independent reflections	intensity decay: 13%
1702 reflections with *I* > 2σ(*I*)	

### Refinement

**Table:**

Refinement on *F*^2^	Secondary atom site location: difference Fourier map
Least-squares matrix: full	Hydrogen site location: inferred from neighbouring sites
*R*[*F*^2^ > 2σ(*F*^2^)] = 0.041	H-atom parameters constrained
*wR*(*F*^2^) = 0.118	*w* = 1/[σ^2^(*F*_o_^2^) + (0.0607*P*)^2^ + 0.5139*P*] where *P* = (*F*_o_^2^ + 2*F*_c_^2^)/3
*S* = 1.05	(Δ/σ)_max_ < 0.001
2275 reflections	Δρ_max_ = 0.36 e Å^−^^3^
155 parameters	Δρ_min_ = −0.47 e Å^−^^3^
0 restraints	Extinction correction: *SHELXL*
Primary atom site location: structure-invariant direct methods	Extinction coefficient: 0.0010 (3)

### Special details

**Table:**

Geometry. All s.u.'s (except the s.u. in the dihedral angle between two l.s. planes) are estimated using the full covariance matrix. The cell s.u.'s are taken into account individually in the estimation of s.u.'s in distances, angles and torsion angles; correlations between s.u.'s in cell parameters are only used when they are defined by crystal symmetry. An approximate (isotropic) treatment of cell s.u.'s is used for estimating s.u.'s involving l.s. planes.
Refinement. Refinement of *F*^2^ against ALL reflections. The weighted *R*-factor *wR* and goodness of fit *S* are based on *F*^2^, conventional *R*-factors *R* are based on *F*, with *F* set to zero for negative *F*^2^. The threshold expression of *F*^2^ > 2σ(*F*^2^) is used only for calculating *R*-factors(gt) *etc*. and is not relevant to the choice of reflections for refinement. *R*-factors based on *F*^2^ are statistically about twice as large as those based on *F*, and *R*- factors based on ALL data will be even larger.

### Fractional atomic coordinates and isotropic or equivalent isotropic displacement parameters (Å^2^)

**Table:**

	*x*	*y*	*z*	*U*_iso_*/*U*_eq_	
Cl1	0.56200 (8)	0.74022 (12)	1.00965 (5)	0.0547 (3)	
Cl4	0.77203 (10)	0.88651 (12)	0.73738 (5)	0.0557 (3)	
Cl2	0.79698 (10)	0.44785 (12)	0.98707 (7)	0.0657 (3)	
Cl3	0.93205 (10)	0.54119 (14)	0.82005 (6)	0.0648 (3)	
O1	0.5602 (2)	0.9949 (3)	0.85728 (14)	0.0479 (5)	
O2	0.5274 (2)	0.7211 (3)	0.81904 (14)	0.0494 (6)	
C5	0.7945 (3)	0.9248 (4)	0.97774 (18)	0.0380 (6)	
H5	0.7385	1.0223	0.9895	0.046*	
C10	0.9019 (3)	0.8836 (4)	1.05363 (19)	0.0410 (7)	
H10A	0.9704	0.8028	1.0405	0.049*	
H10B	0.8517	0.8296	1.0914	0.049*	
C11	0.9836 (3)	1.0369 (4)	1.09459 (19)	0.0434 (7)	
H11A	0.9466	1.1379	1.0655	0.052*	
H11B	0.9643	1.0463	1.1483	0.052*	
C7	0.7734 (3)	0.8387 (4)	0.83817 (19)	0.0424 (7)	
C1	0.4108 (3)	0.9714 (5)	0.8255 (2)	0.0544 (9)	
H1A	0.3719	1.0666	0.7925	0.065*	
H1B	0.3574	0.9574	0.8682	0.065*	
C8	0.8224 (3)	0.6619 (4)	0.8649 (2)	0.0439 (7)	
C6	0.8545 (3)	0.9680 (4)	0.89984 (17)	0.0381 (6)	
H6	0.8212	1.0815	0.8822	0.046*	
C3	0.6241 (3)	0.8363 (4)	0.86189 (19)	0.0404 (7)	
C4	0.6875 (3)	0.7784 (4)	0.94892 (18)	0.0392 (7)	
C9	0.7714 (3)	0.6257 (4)	0.9301 (2)	0.0432 (7)	
C2	0.4067 (4)	0.8167 (6)	0.7776 (3)	0.0729 (12)	
H2A	0.318	0.7555	0.7762	0.088*	
H2B	0.4167	0.8422	0.7232	0.088*	

### Atomic displacement parameters (Å^2^)

**Table:**

	*U*^11^	*U*^22^	*U*^33^	*U*^12^	*U*^13^	*U*^23^
Cl1	0.0427 (4)	0.0648 (5)	0.0619 (5)	−0.0091 (4)	0.0232 (4)	−0.0018 (4)
Cl4	0.0560 (5)	0.0698 (6)	0.0420 (4)	−0.0103 (4)	0.0113 (3)	−0.0032 (4)
Cl2	0.0621 (6)	0.0497 (5)	0.0861 (7)	0.0058 (4)	0.0161 (5)	0.0163 (4)
Cl3	0.0525 (5)	0.0752 (6)	0.0686 (6)	0.0162 (4)	0.0161 (4)	−0.0218 (5)
O1	0.0365 (11)	0.0445 (12)	0.0602 (14)	0.0041 (9)	0.0033 (10)	−0.0058 (10)
O2	0.0357 (11)	0.0508 (13)	0.0591 (14)	−0.0048 (9)	0.0021 (10)	−0.0114 (11)
C5	0.0330 (14)	0.0402 (15)	0.0423 (16)	−0.0014 (12)	0.0113 (12)	−0.0037 (12)
C10	0.0362 (15)	0.0455 (16)	0.0430 (16)	−0.0041 (13)	0.0119 (13)	0.0019 (13)
C11	0.0381 (16)	0.0537 (18)	0.0406 (17)	−0.0047 (13)	0.0127 (13)	−0.0034 (14)
C7	0.0374 (15)	0.0500 (18)	0.0412 (16)	−0.0025 (14)	0.0108 (13)	−0.0043 (13)
C1	0.0339 (16)	0.062 (2)	0.065 (2)	0.0057 (15)	0.0040 (15)	0.0036 (17)
C8	0.0331 (14)	0.0465 (17)	0.0527 (18)	0.0015 (13)	0.0097 (13)	−0.0124 (14)
C6	0.0356 (15)	0.0406 (15)	0.0388 (16)	−0.0018 (12)	0.0086 (12)	−0.0017 (12)
C3	0.0331 (15)	0.0401 (15)	0.0474 (17)	−0.0008 (12)	0.0061 (13)	−0.0062 (13)
C4	0.0329 (14)	0.0427 (16)	0.0442 (16)	−0.0007 (12)	0.0126 (12)	−0.0020 (13)
C9	0.0361 (15)	0.0390 (15)	0.0538 (19)	0.0000 (13)	0.0071 (14)	−0.0007 (14)
C2	0.046 (2)	0.077 (3)	0.085 (3)	0.008 (2)	−0.014 (2)	−0.015 (2)

### Geometric parameters (Å, °)

**Table:**

Cl1—C4	1.751 (3)	C11—H11A	0.97
Cl4—C7	1.754 (3)	C11—H11B	0.97
Cl2—C9	1.700 (3)	C7—C8	1.516 (4)
Cl3—C8	1.701 (3)	C7—C3	1.553 (4)
O1—C3	1.391 (4)	C7—C6	1.560 (4)
O1—C1	1.435 (4)	C1—C2	1.467 (6)
O2—C3	1.397 (4)	C1—H1A	0.97
O2—C2	1.443 (4)	C1—H1B	0.97
C5—C10	1.521 (4)	C8—C9	1.326 (5)
C5—C4	1.559 (4)	C6—C11^i^	1.528 (4)
C5—C6	1.579 (4)	C6—H6	0.98
C5—H5	0.98	C3—C4	1.557 (4)
C10—C11	1.535 (4)	C4—C9	1.517 (4)
C10—H10A	0.97	C2—H2A	0.97
C10—H10B	0.97	C2—H2B	0.97
C11—C6^i^	1.528 (4)		
			
C3—O1—C1	107.2 (2)	H1A—C1—H1B	109
C3—O2—C2	107.3 (3)	C9—C8—C7	108.0 (3)
C10—C5—C4	113.6 (3)	C9—C8—Cl3	127.6 (3)
C10—C5—C6	117.7 (2)	C7—C8—Cl3	124.3 (2)
C4—C5—C6	102.6 (2)	C11^i^—C6—C7	112.9 (2)
C10—C5—H5	107.5	C11^i^—C6—C5	117.9 (2)
C4—C5—H5	107.5	C7—C6—C5	102.1 (2)
C6—C5—H5	107.5	C11^i^—C6—H6	107.8
C5—C10—C11	114.6 (3)	C7—C6—H6	107.8
C5—C10—H10A	108.6	C5—C6—H6	107.8
C11—C10—H10A	108.6	O1—C3—O2	108.7 (2)
C5—C10—H10B	108.6	O1—C3—C7	112.8 (3)
C11—C10—H10B	108.6	O2—C3—C7	114.7 (3)
H10A—C10—H10B	107.6	O1—C3—C4	113.9 (2)
C6^i^—C11—C10	115.3 (3)	O2—C3—C4	113.6 (3)
C6^i^—C11—H11A	108.5	C7—C3—C4	92.5 (2)
C10—C11—H11A	108.5	C9—C4—C3	99.0 (2)
C6^i^—C11—H11B	108.5	C9—C4—C5	108.6 (2)
C10—C11—H11B	108.5	C3—C4—C5	101.0 (2)
H11A—C11—H11B	107.5	C9—C4—Cl1	115.8 (2)
C8—C7—C3	99.0 (2)	C3—C4—Cl1	115.3 (2)
C8—C7—C6	108.6 (3)	C5—C4—Cl1	115.0 (2)
C3—C7—C6	101.2 (2)	C8—C9—C4	107.3 (3)
C8—C7—Cl4	115.9 (2)	C8—C9—Cl2	128.4 (3)
C3—C7—Cl4	115.0 (2)	C4—C9—Cl2	124.2 (2)
C6—C7—Cl4	115.2 (2)	O2—C2—C1	103.4 (3)
O1—C1—C2	103.6 (3)	O2—C2—H2A	111.1
O1—C1—H1A	111	C1—C2—H2A	111.1
C2—C1—H1A	111	O2—C2—H2B	111.1
O1—C1—H1B	111	C1—C2—H2B	111.1
C2—C1—H1B	111	H2A—C2—H2B	109

Symmetry codes: (i) −*x*+2, −*y*+2, −*z*+2.
